# Local control of bone metastases treated with external beam radiotherapy in recent years: a multicenter retrospective study

**DOI:** 10.1186/s13014-021-01940-0

**Published:** 2021-11-20

**Authors:** Kenji Makita, Yasushi Hamamoto, Hiromitsu Kanzaki, Masaaki Kataoka, Shuhei Yamamoto, Kei Nagasaki, Hirofumi Ishikawa, Noriko Takata, Shintaro Tsuruoka, Kotaro Uwatsu, Teruhito Kido

**Affiliations:** 1 Department of Radiology, Ehime University Graduate School of Medicine, 454 Shitsukawa, Toon, Ehime 791-0295 Japan; 2 Department of Radiation Oncology, National Hospital Organization Shikoku Cancer Center, Kou-160, Minami-Umenomoto-Machi, Matsuyama, Ehime 791-0280 Japan; 3 Department of Radiology, Saiseikai Imabari Hospital, 7-1-6 Kita-machi, Imabari, Ehime 799-1592 Japan

**Keywords:** Bone metastases, Local control, External beam radiotherapy, Prognostic factor, Individualized radiotherapy

## Abstract

**Background:**

Over the past decades, remarkable advancements in systemic drug therapy have improved the prognosis of patients with bone metastases. Individualization is required in external beam radiotherapy (EBRT) for bone metastases according to the patient’s prognosis. To establish individualized EBRT for bone metastases, we investigated factors that affect the local control (LC) of bone metastases.

**Methods:**

Between January 2010 and December 2019, 536 patients received EBRT for 751 predominantly osteolytic bone metastases. LC at EBRT sites was evaluated with a follow-up computed tomography. The median EBRT dose was biologically effective dose (BED_10_) (39.0) (range of BED_10_: 14.4–71.7 Gy).

**Results:**

The median follow-up time and median time of computed tomography follow-up were 11 (range 1–123) months and 6 (range 1–119) months, respectively. The 0.5- and 1-year overall survival rates were 73% and 54%, respectively. The 0.5- and 1-year LC rates were 83% and 79%, respectively. In multivariate analysis, higher age (≥ 70 years), non-vertebral bone metastases, unfavorable primary tumor sites (esophageal cancer, colorectal cancer, hepatobiliary/pancreatic cancer, renal/ureter cancer, sarcoma, melanoma, and mesothelioma), lower EBRT dose (BED_10_ < 39.0 Gy), and non-administration of bone-modifying agents (BMAs)/antineoplastic agents after EBRT were significantly unfavorable factors for LC of bone metastases. There was no statistically significant difference in the LC between BED_10_ = 39.0 and BED_10_ > 39.0 Gy.

**Conclusions:**

Regarding tumor-related factors, primary tumor sites and the sites of bone metastases were significant for the LC. As for treatment-related factors, lower EBRT doses (BED_10_ < 39.0 Gy) and non-administration of BMAs/antineoplastic agents after EBRT were associated with poor LC. Dose escalation from BED_10_ = 39.0 Gy did not necessarily improve LC.

## Background

Various tumors frequently result in bone metastases, which are found in 70–85% of advanced cancers diagnosed at the time of death [1]. The incidence rate of bone metastases depends on the primary tumor site and is comparatively higher in breast, prostate, or lung cancers. Bone metastases contribute to only < 20% of the presenting symptoms at diagnosis [2], but can worsen the patient’s quality of life (QOL) with progression.

Radiotherapy is useful for pain relief of bone metastases. In terms of pain relief and adverse events, single-fraction external beam radiotherapy (EBRT) of 8 Gy is comparable with 30 Gy in 10 fractions or 20 Gy in five fractions [3]. Many guidelines for managing of bone metastases recommend single-fraction EBRT of 8 Gy for pain relief of uncomplicated bone metastases. However, despite no significant difference in the duration of pain relief between single-fraction and fractionated EBRT, the period of pain relief tends to be longer after fractionated EBRT [4]. The incidence rate of retreatment was lower in fractionated EBRT than in single-fraction EBRT [5].

In recent years, the significant progress in systemic and supportive therapies has improved the expected prognosis of patients with advanced cancers [6, 7]. Thus, local control (LC) of bone metastases becomes more important for patients with a favorable prognosis. To individualize the EBRT for bone metastases, knowledge of factors associated with LC is essential; however, factors affecting LC of bone metastases (tumor-, treatment-, and patient-related factors) have not been fully investigated. In this study, we aimed to determine the factors affecting the LC in bone metastases receiving EBRT.

## Methods

**Fig. 1:**
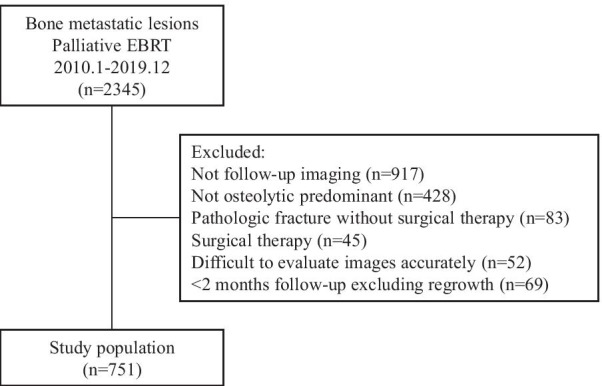
Study flow diagram. *EBRT* external beam radiotherapy

Between January 2010 and December 2019, 1750 patients with 2345 bone metastatic lesions were treated with EBRT by three-dimensional conformal radiotherapy in three institutions: (a) cancer center (n = 1514), (b) university hospital (n = 594), and (c) community hospital (n = 237). It is often difficult to evaluate the tumor response to EBRT in predominantly osteoplastic bone metastases on computed tomography (CT) image because it was difficult to differentiate regrowth of predominantly osteoplastic bone metastases from reparative ossification after radiotherapy. Therefore, only predominantly osteolytic bone metastases were examined. A total of 536 patients with 751 predominantly osteolytic bone metastatic lesions were followed up with CT ≥ 2 months (including regrowth in < 2 months) after EBRT treatment. The LC of EBRT sites in these patients was evaluated in this retrospective analysis (Fig. 1).

### Radiotherapy

The doses of EBRT were determined at the discretion of each physicist and institution; 30 Gy in 10 fractions was the most frequently used dosage. EBRT was performed with 6- to 10-MV X-ray of linear accelerators, and the doses of the target volumes were prescribed to be ≥ 90% of the EBRT dose, in principle. The biologically effective dose (BED) was calculated to compare the various fractionated schedules. The BED_10_ (BED calculated using an α/β of 10 Gy) was calculated by *nd* (1 + *d*/(α/β)), where *d* is the fraction dose, *n* is the number of fractions, and α/β is 10 Gy.

### Effectiveness assessment

The primary endpoint of this study was the LC of EBRT sites for bone metastases. The extracted outcomes were classified according to the presence or absence of local recurrence, or local regrowth, at the EBRT sites of bone metastases. Local control was defined as when the irradiated bone metastases were stable or shrunk. Two observers (a radiologist and a radiation oncologist) were blinded to the follow-up information and outcomes during the evaluation of the images.

### Statistical analyses

The survival duration and the LC period of EBRT sites were calculated from the start of palliative EBRT. The Kaplan–Meier method was used to generate LC and overall survival (OS) curves. We assessed the predictive factors associated with LC rates of EBRT sites using univariate and multivariate Cox proportional hazards models to determine hazard ratios (HRs), including 95% confidence intervals (CIs) and p-value. Variables included in the multivariate models had a p-value of < 0.1 in the univariate analysis. Statistical analyses were performed using the JMP software (JMP version 14.3.0; SAS Institute, Cary, NC, USA).

## Results

Data from 536 patients (male/female = 315/221; age, median [range]: 66 [12–90] years) with 751 lesions were included in the analysis dataset. The median follow-up time and median time of CT follow-up were 11 (range: 1–123) months and 6 (range: 1–119) months, respectively. Details of the lesion characteristics are shown in Table 1. The median EBRT dose was BED_10_ = 39.0 Gy (= 30 Gy in 10 fractions). The other fraction schedules, in sequential order, for EBRT of BED_10_ (= fraction schedules) were as follows: 14.4 Gy (= 1 × 8 Gy), 28.0 Gy (= 5 × 4 Gy), 30.0 Gy (= 4 × 5 Gy), 31.2 Gy (= 10 × 2.5 Gy), 46.9–56.2 Gy (= 15–18 × 2.5 Gy), 42.9–58.8 Gy (= 11–15 × 3 Gy), 50.4–60.0 Gy (= 21–25 × 2 Gy), 39.7 Gy (= 5 × 4 Gy + 3 × 3 Gy), 47.2 Gy (= 5 × 4 Gy + 8 × 2 Gy), and 71.7 Gy (= 3 × 3 Gy + 25 × 2 Gy).

**Table 1:** Characteristics of lesions

Characteristic	No. of lesions	%
*Age*		
< 70 years	504	67.1
≥ 70 years	247	32.9
*Sex*		
Male	447	59.5
Female	304	49.5
*Primary tumor sites*		
Lung	248	33.0
Breast	137	18.2
Head and neck	53	7.1
Esophagus	15	2.0
Hepatobiliary/pancreatic	95	12.7
Kidney/ureter	74	9.9
Colorectal	31	4.1
Gynecological	19	2.5
Sarcoma/melanoma/mesothelioma	18	2.4
Others	61	8.1
*EBRT sites*		
Vertebral	445	59.2
Pelvis	182	24.2
Rib	65	8.7
Others	59	7.9
*Bone cortex destruction*		
Yes	557	74.2
No	194	25.8
*EBRT dose (BED*_*10*_*)*		
Median: 39.0 (14.4—71.7)		
14.4	22	2.9
> 14.4, < 39.0	84	11.2
39	434	57.8
> 39.0	211	28.1
*Post-EBRT BMAs*		
Yes	460	61.3
No	291	38.7
*Pre-EBRT ATs*		
Yes	408	54.3
No	343	45.7
*Post-EBRT ATs*		
Yes	518	69.0
No	233	31.0

*EBRT* External beam radiotherapy, *BMAs* bone modifying agents, *ATs* antineoplastic agents, *BED* biologically effective dose

### Overall survival (OS) and local control (LC) of the external beam radiotherapy (EBRT) sites

**Fig. 2:**
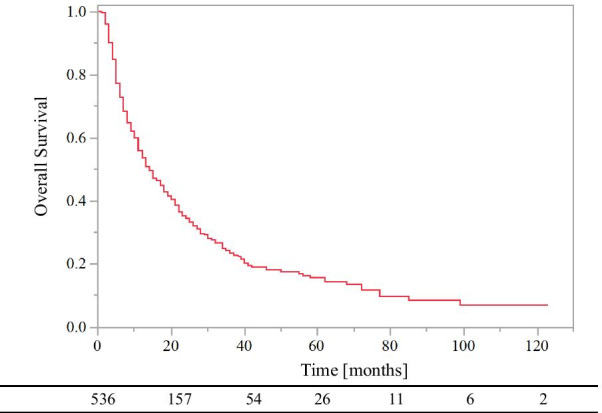
Overall survival of all patients

The 0.5- and 1-year OS rates were 73% and 54%, respectively (Fig. 2). The 1-year OS rates from January 2010 to December 2016 and from January 2017 to December 2019 were 52% and 58%, respectively (p = 0.026, log-rank).

**Fig. 3:**
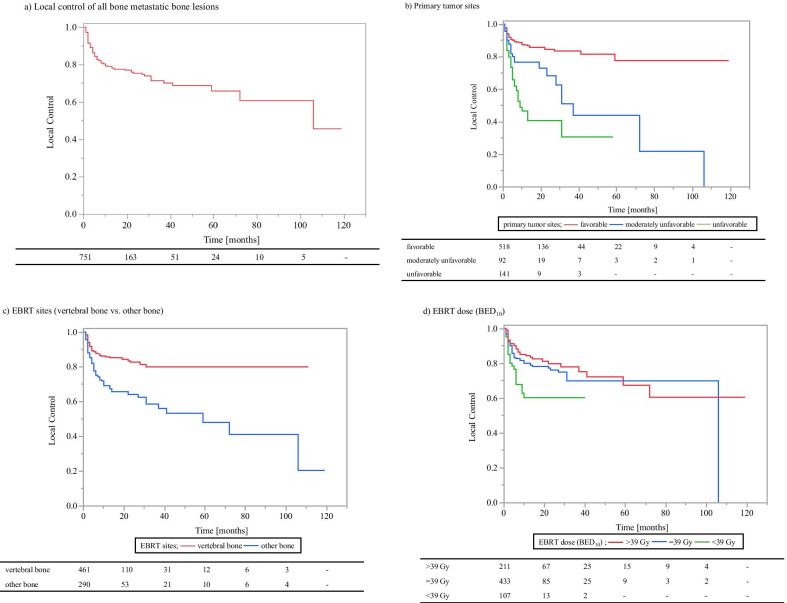
Local control of bone metastases. **a** Local control of all bone metastatic bone lesions. **b** Primary tumor sites (favorable group vs. moderately unfavorable group vs. unfavorable group; favorable group: head and neck, lung/mediastinal, breast, gastric, gynecologic, prostate, bladder, and skin cancers; moderately unfavorable group: kidney/ureter and non-epithelial cancers; unfavorable group: esophageal, colorectal, and hepatobiliary/pancreatic cancers). **c** EBRT sites (vertebral bone vs. other bone). **d** EBRT dose (BED_10_) (< 39.0 Gy vs. 39.0 Gy vs. > 39.0 Gy). *BED* Biologically effective dose, *EBRT* external beam radiotherapy

Local recurrence was observed in 19.6% (147/751) of EBRT sites, and the median time to recurrence was 3 (range: 1–106) months. The 0.5- and 1-year LC rates of EBRT sites were 83% and 79%, respectively (Fig. 3a). The OS rate of patients with local regrowth and those without local regrowth was 60% and 76%, respectively at 0.5-year, and 38% and 58%, respectively at 1-year (p = 0.001, log-rank). In addition, the 0.5- and 1-year OS rates after the local regrowth were 33% and 19%, respectively.

### LC according to primary tumor sites

Our study's primary tumor sites were classified into three groups based on reported radiosensitivity [8] and 1-year LC rates (Table 2). Esophageal cancer, colorectal cancer, and hepatobiliary/pancreatic cancer were classified as the unfavorable group. Kidney/ureter cancer and non-epithelial tumors (sarcoma/melanoma/mesothelioma) were classified as the moderately unfavorable group. The remaining (i.e., lung cancer, breast cancer, head and neck cancer, gastric cancer, genitourinary cancer, and skin cancers) were classified as the favorable group.

**Table 2:** Risk group classification of primary tumor sites according to local control rates

Primary tumor sites	6 months (%)	12 months (%)	36 months (%)
*Unfavorable group*			
Esophagus	61	49	49
Hepatobiliary/pancreatic	54	49	38
colorectal	85	43	21
*Moderately unfavorable group*			
Kidney/ureter	75	75	48
Sarcoma/melanoma/mesothelioma	84	84	28
*Favorable group*			
Lung	83	79	76
Breast	98	98	98
Head and neck	88	88	68
Gynecological	100	100	86
Others	94	86	86

The 0.5- and 1-year LC rates were 62% and 47% for the unfavorable, 77% and 77% for the moderately unfavorable, and 89% and 87% for the favorable groups, respectively (Fig. 3b). On univariate analysis, LC rates were significantly lower in the unfavorable group compared with the moderately unfavorable group (HR 1.84, 95% CI 1.15–2.95, p = 0.011 [Table 3]) and significantly higher in the favorable group compared with the moderately unfavorable group (HR 0.40, 95% CI 0.26–0.64, p < 0.001 [Table 3]).

**Table 3:** Local control rates after EBRT and results of univariate and multivariate analyses

		0.5-year (%)	1-year (%)	Univariate analysis		Multivariate analysis	
				HR (95% CI)	*P*	HR (95% CI)	*P*
Age	< 70 years vs. ≥ 70 years	89 vs. 71	84 vs. 69	2.32 (1.68–3.21)	< 0.001	2.34 (1.62–3.40)	< 0.001
Sex	Female vs. male	88 vs. 78	86 vs. 73	1.92 (1.35–2.72)	< 0.001	1.15 (0.76–1.73)	0.514
Primary tumor sites	Moderately unfavorable vs. favorable	77 vs. 89	77 vs. 87	0.40 (0.26–0.64)	< 0.001	0.49 (0.29–0.81)	0.006
	Moderately unfavorable vs. unfavorable	77 vs. 62	77 vs. 47	1.84 (1.15–2.95)	0.011	2.28 (1.34–3.86)	0.002
EBRT sites	Vertebral bone vs. other bone	88 vs. 75	86 vs. 69	2.34 (1.69–3.25)	< 0.001	1.78 (1.24–2.57)	0.002
EBRT dose (BED_10_)	≥ 39.0 Gy vs. < 39.0 Gy	85 vs. 68	82 vs. 60	2.02 (1.34–3.05)	0.001	2.08 (1.31–3.30)	0.002
Post-EBRT BMAs	Yes vs. no	88 vs. 74	86 vs. 67	2.49 (1.79–3.46)	< 0.001	1.94 (1.34–2.83)	< 0.001
Post-EBRT ATs	Yes vs. no	88 vs. 70	84 vs. 65	2.41 (1.73–3.36)	< 0.001	1.58 (1.08–2.31)	0.018
Bone cortex destruction	Yes vs. no	80 vs. 90	77 vs. 84	0.70 (0.46–1.05)	0.083	0.67 (0.42–1.06)	0.084
Pre-EBRT ATs	Yes vs. no	85 vs. 80	80 vs. 78	1.24 (0.89–1.71)	0.198	–	–

*EBRT* External beam radiotherapy, *BMAs* bone modifying agents, *ATs* antineoplastic agents, *BED* biologically effective dose

### LC according to EBRT sites

The 0.5- and 1-year LC rates after EBRT were 88% and 86% for vertebral metastases and 75% and 69% for non-vertebral bone metastases (Fig. 3c). On univariate analysis, the LC rates were significantly lower in the non-vertebral bone metastases compared with the vertebral bone metastases (HR 2.34, 95% CI 1.69–3.25, p < 0.001 [Table 3]).

### LC according to EBRT doses (BED_10_)

The 0.5- and 1-year LC rates were 68% and 60% for BED_10_ < 39.0 Gy, 83% and 80% for BED_10_ = 39.0 Gy, and 88% and 84% for BED_10_ > 39.0 Gy (Fig. 3d). The LC rate was significantly lower for BED_10_ < 39.0 Gy than BED_10_ ≥ 39.0 Gy (HR 2.02, 95% CI 1.34–3.05, p = 0.001) on univariate analysis. In addition, there were statistically significant differences in LC rates between BED_10_ < 39.0 Gy and BED_10_ = 39.0 Gy (HR 1.87, 95% CI 1.22–2.87, p = 0.004). In contrast, no statistically significant differences were found between BED_10_ = 39.0 Gy and BED_10_ > 39.0 Gy (HR 1.26, 95% CI 0.86–1.87, p = 0.240).

### Dose escalation from BED_10_ = 39.0 Gy and LC according to primary tumor sites and metastatic sites

#### According to primary tumor sites

For the unfavorable group of primary tumor sites, the 1-year LC rates of BED_10_ = 39.0 and BED_10_ > 39.0 Gy were 46% and 59%, respectively (HR 1.44, 95% CI 0.77–2.70, p = 0.251). For the moderately unfavorable group of primary tumor sites, the 1-year LC rates of BED_10_ = 39.0 and BED_10_ > 39.0 Gy were 78% and 86%, respectively (HR 1.75, 95% CI 0.70–4.36, p = 0.232). For the favorable group of primary tumor sites, the 1-year LC rates of BED_10_ = 39.0 and BED_10_ > 39.0 Gy were 90% and 94%, respectively (HR 1.16, 95% CI 0.63–2.13, p = 0.631). Dose escalation from BED_10_ = 39.0 Gy did not improve LC significantly, especially for the favorable group.

#### According to metastatic sites

For vertebral metastases, the 1-year LC rates were 86% and 91% for BED_10_ = 39.0 and BED_10_ > 39.0 Gy, respectively (HR 1.17, 95% CI 0.64–2.16, p = 0.615). For non-vertebral bone metastases, the 1-year LC rates were 69% and 80% for BED_10_ = 39.0 and BED_10_ > 39.0 Gy, respectively (HR 1.59, 95% CI 0.96–2.62, p = 0.070). Dose escalation from BED_10_ = 39.0 Gy tended to improve LC for non-vertebral bone metastases.

The incidence of high dose EBRT (BED_10_ ≥ 39.0 Gy) was not different according to metastatic sites (vertebral bone, 86.6%; non-vertebral bone, 85.3%; p = 0.619, chi-square test). The proportion of bone metastases from the unfavorable and moderately unfavorable primary tumor sites was higher in non-vertebral bone compared to vertebral bone metastases (39.0% vs. 26.0%, p < 0.001, chi-square test).

### LC according to other factors

Male, higher age (≥ 70 years), non-administration of BMAs/antineoplastic agents (ATs, including hormone therapy) after EBRT (post-EBRT BMAs/ATs), and the destruction of cortical bone were statistically significant unfavorable factors for LC on univariate analysis (Table 3). In principle, cytotoxic chemotherapy, biotherapy, and immune-checkpoint inhibitor were not used in concurrent combination therapy with EBRT. The administration of ATs before EBRT (pre-EBRT ATs) was not a significant factor for LC on the univariate analysis (Table 3).

### Multivariate Cox regression analysis

On multivariate analysis, higher age (≥ 70 years), bone metastases from unfavorable/moderately unfavorable groups of primary tumor sites, non-vertebral bone metastases, EBRT dose of BED_10_ < 39.0 Gy, and non-administration of BMAs/ATs after EBRT were significantly unfavorable independent factors for LC (Table 3).

## Discussion

Among the patients who received EBRT to bone metastases in recent years, approximately half of them were estimated to survive for 1 year. Approximately 80% of the bone metastases receiving EBRT in clinical practice were estimated to achieve LC for 1 year in our practice. LC rates after EBRT were satisfactory for the majority of patients. LC rates were influenced by some tumor-, treatment-, and patient-related factors. Regarding tumor-related factors, both primary tumor sites and sites of bone metastases (vertebral bone vs. non-vertebral bone) were associated with LC. Regarding treatment-related factors, lower doses of EBRT (BED_10_ < 39.0 Gy) and non-administration of BMAs/ATs after EBRT were associated with poor LC. Interestingly, dose escalation from a BED_10_ = 39.0 Gy did not necessarily lead to improvement of LC. Regarding patient-related factors, higher age (≥ 70 years) seemed to be associated with poor LC.

Recently, long-term LC of bone metastases is required for patients with relatively good prognosis. In contrast, some patients still have a poor prognosis despite advancements in systemic therapy. Katagiri reported that the 3-year OS rate of patients with bone metastases was 23% [9], which is similar to that in our study (24%). It is noteworthy that one-fifth of the patients with bone metastases were estimated to survive 3 years or more. These patients may need more aggressive EBRT for bone metastases.

For bone metastases from unfavorable and moderately unfavorable primary tumor sites, approximately half progressed within 1–3 years after EBRT. In addition, the prognosis of patients with unfavorable and moderately unfavorable primary tumor sites was generally poorer. The 1-year OS rate of these patients was only 41% (data not shown) in our present study. Therefore, the majority of patients with unfavorable and moderately unfavorable primary tumor sites may not experience a decrease in QOL due to bone metastases. However, some patients with unfavorable and moderately unfavorable primary tumor sites survive for a relatively long time and experience a decrease in QOL. Considering the poor LC rates of bone metastases from unfavorable and moderately unfavorable primary tumor sites, EBRT with a median BED_10_ = 39.0 Gy seemed to be insufficient for some long-term survivors who have unfavorable and moderately unfavorable primary tumor sites. More aggressive EBRT, such as stereotactic body radiotherapy (SBRT) or heavy ion therapy, may be better performed for these patients. Some studies have shown that SBRT for bone metastases from renal cell cancer, sarcoma, and melanoma (which were included in the moderately unfavorable group of primary tumor sites) achieved good LC of the irradiated sites [10–12]. However, bone metastases from hepatocellular carcinoma and colorectal cancer (which were included in the unfavorable group of primary tumor sites) were difficult to control, regardless of using SBRT [13, 14]. Although further studies are needed, it may be difficult to control bone metastases from unfavorable primary tumor sites. LC rates of bone metastases from favorable primary tumor sites were generally satisfactory, with a 1-year LC rate of approximately 90%. Although stereotactic radiosurgery seems to further increase LC [15], conventional EBRT seems to be suitable for the majority of patients with favorable primary tumor sites.

The site of bone metastases was associated with the LC in our study. LC rates of vertebral metastases were higher compared with those of other bone metastases. The EBRT site of bone metastases is considered to occur through a multistep process involving interactions between cancer cells and normal host cells [16]. Vertebral metastases often occur via Batson’s vertebral venous plexus, which bypasses the lung and liver [17]. Because of the lack of checkpoints such as the lung and liver, cancer cells in vertebral metastases may have slightly different characteristics from those in non-vertebral bone metastases. It remains unclear why LC of vertebral metastases was better, but this could be one of the possible explanations for the difference in radiosensitivity between vertebral metastases and non-vertebral bone metastases. It was true that the proportion of bone metastases from favorable primary tumor sites were higher in vertebral metastases compared to non-vertebral metastases; multivariate analysis showed vertebral metastases were independently significant favorable factors for local control. Therefore, we think that vertebral metastases were favorable factors for local control.

Regarding treatment-related factors, BED_10_ < 39.0 Gy and non-administration of BMAs/ATs after EBRT were unfavorable factors for LC of bone metastases in our present study. Although it has been reported that LC of bone metastases tends to be dose dependent [18–21], there was no significant difference in LC rates between BED_10_ = 39.0 Gy and BED_10_ > 39.0 Gy. Especially for bone metastases from favorable primary tumor sites, dose escalation from BED_10_ = 39.0 Gy seemed to have little effect on LC rates (39.0 Gy, 90% at 1 year; > 39.0 Gy, 94% at 1 year). In contrast, dose escalation from BED_10_ = 39.0 Gy tended to improve LC in non-vertebral bone metastases (39.0 Gy, 69% at 1 year; > 39.0 Gy, 80% at 1 year). Further studies are needed to identify the bone metastases that will benefit from more aggressive EBRT.

Another treatment-related factor for LC of bone metastases was the administration of systemic drug therapy after EBRT. Non-administration of BMAs/ATs after EBRT was an unfavorable factor for LC of bone metastases. Several studies have shown that a combination of EBRT and BMAs improved effectiveness compared with EBRT alone or BMAs alone [22–24]. The response rate of bone metastases to ATs was 8–59% [25–34]. BMAs and ATs after EBRT seemed to potentially enhance the effect of EBRT on bone metastases. Administration of BMAs and ATs after EBRT seemed to be useful in patients with good prognosis.

This study has some limitations owing to its retrospective nature. First, osteoplastic bone metastases were excluded from this study because it is often difficult to evaluate the LC. As a result, many bone metastases from prostate cancer, which are often osteoplastic, were excluded from our present study. Second, the number of each primary tumor site was relatively small; hence, there is a possibility that the LC according to the primary tumor site was not evaluated accurately. There remained the possibility that dose escalation in EBRT from BED_10_ > 39.0 Gy may be beneficial for bone metastases with a comparatively good prognosis and radio-resistant nature. For example, EBRT of > 50 Gy improved LC compared with EBRT of < 50 Gy in bone metastases from differentiated thyroid cancer [19]. Third, there might be a selection bias in the determination of EBRT doses because many attending radiation oncologists were involved in the management of patients due to the multicenter and long-term study design. Finally, detailed information on pain was unavailable from the clinical records of many patients. Therefore, the relationship between regrowth of bone metastases and pain could not be evaluated. Although patients with poor prognoses need only short-term pain control, patients with good prognoses are likely to need long-term local control of EBRT sites. Knowledge of factors that affects LC of bone metastases is essential for long-term local control. We believe that knowledge of factors affecting LC of bone metastases is the basis of individualized radiotherapy.

Tumor-, treatment-, and patient-related factors influenced the LC of bone metastases after EBRT. For tumor-related factors, not only primary tumor sites but also sites of bone metastases are significant for the LC. After conventional EBRT with the median dose of BED_10_ = 39.0 Gy, LC rates of bone metastases were favorable for many cancers, whereas they were lower for esophageal cancer, colorectal cancer, hepatobiliary/pancreatic cancer, kidney/ureter cancer, and sarcoma/melanoma/mesothelioma. Vertebral metastases showed significantly better LC compared with metastases of other bones. As for treatment-related factors, lower EBRT doses (BED_10_ < 39.0 Gy) and non-administration of BMAs/ATs were associated with poor LC. Dose escalation in EBRT from a BED_10_ = 39.0 Gy did not necessarily improve LC. In addition to the predicted prognosis, these results should be considered for the individualization of EBRT for bone metastases.

## Data Availability

Not applicable.

## Abbreviations

ATs: Antineoplastic agents
BED_10_: Biologically effective dose
BMAs: Bone-modifying agents
CIs: Confidence intervals
CT: Computed tomography
EBRT: External beam radiotherapy
HRs: Hazard ratios
LC: Local control
OS: Overall survival
QOL: Quality of life
